# Design Parameter Optimization of a Silicon-Based Grating Waveguide for Performance Improvement in Biochemical Sensor Application

**DOI:** 10.3390/s18030781

**Published:** 2018-03-05

**Authors:** Yoo-Seung Hong, Chun-Hyung Cho, Hyuk-Kee Sung

**Affiliations:** 1 School of Electronic and Electrical Engineering, Hongik University, Seoul 04066, Korea; yoosing87@gmail.com; 2 Department of Electronic & Electrical Engineering, College of Science and Technology, Hongik University, Sejong 30016, Korea; chcho@hongik.ac.kr

**Keywords:** diffraction gratings, grating waveguide, refractive index sensor, biological sensing and sensors, optical sensing and sensors

## Abstract

We performed numerical analysis and design parameter optimization of a silicon-based grating waveguide refractive index (RI) sensor. The performance of the grating waveguide RI sensor was determined by the full-width at half-maximum (FWHM) and the shift in the resonance wavelength in the transmission spectrum. The transmission extinction, a major figure-of-merit of an RI sensor that reflects both FWHM and resonance shift performance, could be significantly improved by the proper determination of three major grating waveguide parameters: duty ratio, grating period, and etching depth. We analyzed the transmission characteristics of the grating waveguide under various design parameter conditions using a finite-difference time domain method. We achieved a transmission extinction improvement of >26 dB under a given bioenvironmental target change by the proper choice of the design procedure and parameters. This design procedure and choice of appropriate parameters would enable the widespread application of silicon-based grating waveguide in high-performance RI biochemical sensor.

## 1. Introduction

Optical refractive index (RI) sensors that measure the change in RI have been proven useful in successfully developing a biochemical sensor that measures biomolecular interactions [1,2,3,4]. These optical RI sensors exhibit significant advantages over conventional mechanical and electrochemical sensors. These advantages include no fluorescent labeling, high throughput, and better sensitivity [5,6,7,8,9]. Several approaches to quantify the RI change of a target layer, which is induced by the variation in the sensing environment, have been proposed and found to be effective in achieving a high-precision RI sensor. The various optical RI sensors include surface plasmon resonance (SPR) [10], ring resonator [11], long-period fiber grating [12], grating coupler [13], grated waveguide [14], and metallic photonic crystal sensors [15]. The proposed techniques utilize the resonant frequency shift of a transmission spectral profile that occurs when a fraction of the guided mode interacts with the change in the RI of the waveguide surface layer. The silicon-based grating waveguide RI sensor possesses two major advantages over other types of optical RI sensors. These advantages are high-resolution performance in detecting changes in the RI and mass production capability regarding device fabrication. The former advantage is due to the shift of a sharp fringe in the transmission spectrum near the stopband edge of the grating [14], and the latter is due to a solid and matured processing technology of silicon (Si)-based electrical/optical device fabrication such as the standard complementary metal-oxide-semiconductor (CMOS) process [16]. Several pieces of research successfully demonstrated the capability of Si-based grating waveguide sensor in biochemical applications [17,18,19]. We recently proposed a compact and inexpensive biochemical sensor prototype using the Si-based grating waveguide to selectively detect and quantify the multivalent binding of proteins from a monovalent binding [19], which is known to be a critical step for better understanding of fundamental mechanisms in the immune system, cancer, and thrombosis [20,21,22]. The proposed biochemical sensor consists of a typical silicon-on-insulator (SOI) bottom layer, a silicon-based grating waveguide core, and a hydrogel functional layer on top of the grating waveguide. We calculated the dynamic range of the effective RI of the waveguide and demonstrated the feasibility of silicon-based grating waveguide sensors assisted by a functional hydrogel layer in addition to determining a design principle for it. Although our previous work and other studies on the Si-based grating waveguide sensor exhibit feasibility and high performance in detecting the changes in the RI of the target layer, the detailed analysis of the sensor performance considering the grating structure parameters and its design procedures have not been demonstrated yet.

In this paper, we propose a method to achieve maximum transmission extinction by choosing appropriate grating waveguide parameters, which are the duty ratio, grating period, and etching depth. We calculated the transmission characteristics of the grating waveguide sensor using a finite-difference time domain (FDTD) method. Here, we defined the transmission extinction as an important figure-of-merit (FOM) because it reflects both the resonance wavelength shift and full-width at half-maximum (FWHM) performance in the transmission spectrum of the RI sensor. We determined the relationship between the parameters and the sensor performance, and found that among the three parameters, the etching depth has the greatest effect on transmission extinction. We established the design guideline to determine the proper grating parameters based on the parameter vs. performance relationship. By using the procedure and with the optimization of the parameters, we achieved 49.2 dB of transmission extinction, which is an improvement of >26 dB when compared with the value attained without careful parameter selection.

## 2. Principles

**Figure 1:**
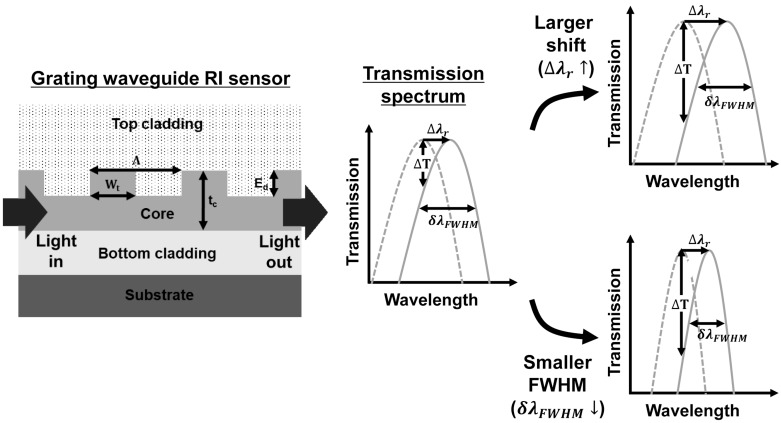
A grating waveguide refractive index (RI) sensor and its sensing principle. The figure set on right side depicts the dependence of transmission extinction ΔT on a resonance wavelength shift (top right) and FWHM (bottom right); $W_{t}$: width of tooth grating, Λ: grating period, $t_{c}$: thickness of waveguide core, $E_{d}$: etching depth, $\Delta \lambda_{r}$: resonance wavelength shift, $\delta \lambda_{FWHM}$: full-width at half-maximum, ΔT: transmission extinction.

Several sensor platforms such as transverse magnetic (TM) field and SPR sensor platforms have been proposed to increase the amount of transmission spectrum shift occurring due to the bioenvironmental target change [23,24]. The ratio of the evanescent field to the guiding field in a TM field sensor is larger than that in a transverse electric (TE)-field type sensor, so that the effect of the change in RI can be significantly enhanced, resulting in a high resolution of the RI sensor [23]. An SPR sensor utilizes the enhancement in the interaction of the surface wave with the target material/phenomenon, which also provides high-resolution capability [24]. These RI optical sensors can be widely applied to biochemical sensors owing to their high-resolution performances. The high-resolution performance is attributed to the large transmission spectrum shift due to the change in the resonance wavelength. Another important FOM of the optical RI sensor is the FWHM of the transmission spectrum. Resonator-type RI sensors with a high-quality factor (Q-factor) can provide high-resolution performance in measuring a tiny change in the effective RI of the optical waveguide [25]. Typically, the performance of the resonator-type optical RI sensor is evaluated either by the resonance wavelength shift, $\Delta \lambda_{r}$, or the FWHM, $\delta \lambda_{FWHM}$. Figure 1 shows a grating waveguide RI sensor and its sensing principle. Light that travels through the grating waveguide core is diffracted by the grating structure. The output transmission spectrum exhibits a resonance for which the wavelength shifts owing to the change in the RI of the top cladding layer. The set of figures on the right side of Figure 1 depict the effects of $\Delta \lambda_{r}$ and $\delta \lambda_{FWHM}$ on the transmission spectrum. In the top right part of Figure 1, a large transmission extinction ΔT is achieved by a large resonance wavelength shift. In the bottom right part of Figure 1, a large transmission extinction ΔT is achieved by a small FWHM. In designing high-performance optical RI sensors, both the resonance wavelength shift $\Delta \lambda_{r}$ and FWHM $\delta \lambda_{FWHM}$ should be simultaneously considered to increase the transmission extinction ΔT. Herein, we propose the complete optimization of the design structure of the silicon-based grating waveguide for high-performance RI optical sensors used in biochemical applications by considering both the effect of the resonance wavelength shift $\Delta \lambda_{r}$ and FWHM $\delta \lambda_{FWHM}$.

**Figure 2:**
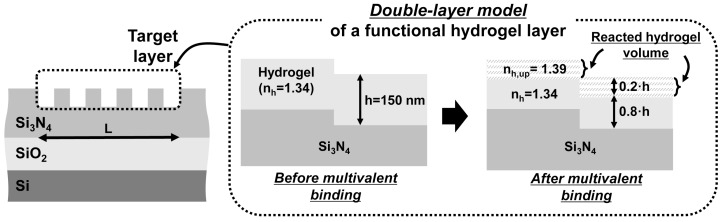
A silicon-based grating waveguide refractive index (RI) sensor and conceptual diagram showing the RI change of a functional hydrogel layer using a double-layer model [19,26]. The inset shows the shape of the hydrogel layer and grating waveguide that corresponds to a single tooth-groove period. L: grating length, h: hydrogel layer thickness, $n_{h}$: RI of the hydrogel layer before the multivalent binding or RI of the non-reacted hydrogel volume after the multivalent binding, $n_{h,up}$: RI of the reacted hydrogel volume after the multivalent binding.

Figure 2 shows the silicon-based grating waveguide sensor platform for biochemical applications, which is similar to that discussed in [19]. The proposed biochemical sensor consists of a typical SOI bottom layer, a silicon-based grating waveguide core, and a target layer on top of the grating waveguide. The waveguide core with grating structures provides Fabry–Perot resonances of the Bloch modes. The resultant transmission spectral profile exhibits multiple resonance peaks due to optical interference of the guided modes in the grating waveguide. In our analysis, a functional hydrogel layer was used as the target layer, as shown in the inset of Figure 2, because it can provide a highly sensitive RI change when biochemical interaction occurs in the layer. We performed all calculations based on the shape of the hydrogel-waveguide interface assuming that the deposited hydrogel layer penetrates the waveguide groove structure and fills the groove structure completely, as shown in the inset of Figure 2. It is known that receptor proteins in the hydrogel layer exhibit multivalent bindings with injected target proteins, which result in significant change in the RI of the hydrogel layer by local deswelling. We used a double-layer model for the functional hydrogel layer to adopt the results in [26]. In the double-layer model, the RI of a part of the upper portion (=20%) of the layer changes to 1.39 ($n_{h,up}$ = 1.39) and that of the other portion (80%) remains at 1.34 ($n_{h}$ = 1.34) when the target bio-interaction such as multivalent binding of proteins occurs [26]. In our previous work on the grating waveguide sensor performance [19], we varied the top portion of the hydrogel layer that experiences the RI change (i.e., functional volume ratio) between 15–25%, because it can vary with several changing factors such as temperature, receptor-protein pairs, and hydrogel composition. The optimized waveguide parameters can be determined using the method provided in the manuscript for each case of the different biological environment. The hydrogel layer thickness is set at 150 nm because the thickness exhibits the maximum change in the effective RI of the waveguide core [19]. We applied this functional hydrogel model as the target layer and analyzed the resonance shift $\Delta \lambda_{r}$, FWHM $\delta \lambda_{FWHM}$, and transmission extinction ΔT of the grating waveguide sensor.

The change in the RI of the target layer affects the effective change in the RI of the waveguide core, resulting in a change in the transmission characteristic. The waveguide core consists of Si_3_N_4_ ($n_{core}=1.979$) on top of a SiO_2_ bottom cladding layer $\left(n_{cladding}=1.444\right)$ with a Si substrate ($n_{substrate}=3.476$) at 1550-nm wavelength [27,28]. Initially, the thickness of the waveguide core ($t_{c}$) is set at 275 nm, the grating period (Λ) is 490 nm, the duty ratio (R = $\frac{W_{t}}{\Lambda }$) is 0.5, and the number of the grating structure elements is 200. The duty ratio is defined as the ratio of the tooth grating width $W_{t}$ to the grating period Λ. The total grating length L is 98 μm. The FWHM becomes smaller when the grating length L increases because of the increased intra cavity effect, and it is represented by [17]

(1) $$\delta \lambda_{FWHM}=\frac{\lambda_{r}^{2}}{2\pi LN_{g}},$$

where $N_{g}$ is the group index of the waveguide core and $\lambda_{r}$ is the resonance wavelength. As shown in Figure 1, a smaller FWHM is preferred for achieving a large transmission extinction ΔT. Although a smaller FWHM can be achieved by increasing the grating length L as in (1), the grating length may not be varied across a wide range because the waveguide transmission loss increases with L. The grating length may also be related to the size of the other sensor components such as the detector, electronic circuits, and optical coupler/divider for an on-chip integration platform. Consequently, we evaluated the dependence of the transmission extinction ΔT on the three major grating parameters, which are the duty ratio, grating period Λ, and etching depth $E_{d}$, under a fixed grating length L of 98 μm. Based on the performance evaluation and its dependence on the grating parameters, we have provided an optimization method and proposed the design procedure of the silicon-based grating waveguide sensor. We performed a numerical investigation of the grating waveguide using a 2-D FDTD method and a finite-difference method for TE mode using software from Lumerical Solutions, Inc., London, UK. We used a Gaussian pulse with a center wavelength of 1550 nm and a span of 400 nm. The waveguide transmission characteristics are calculated by carrying out fast Fourier transform (FFT) with 80,000 sample points. We incorporated a 2-D FDTD simulation window of 115 μm in the *x*-direction (propagation direction) and 5 μm in the *y*-direction (cross-section direction) using perfectly matched layers under boundary conditions. To obtain accurate transmission in the grating waveguide, a mesh grid size of ∆x = 52.9 nm in the *x*-direction, ∆y = 6 nm in the *y*-direction, and a time-step size of $\Delta t=1.3\cdot 10^{-17}$ s was used in the FDTD simulation.

**Figure 3:**
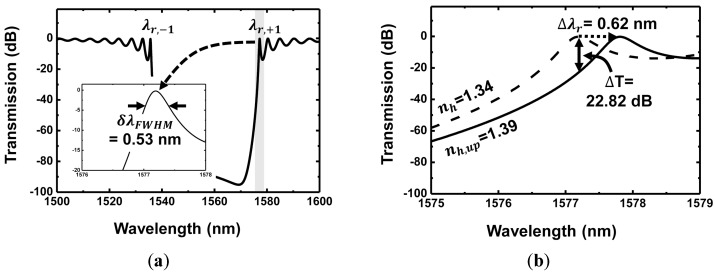
(**a**) Transmission spectrum of a grating waveguide showing the stopband and narrow FWHM $\delta \lambda_{FWHM}$ at $\lambda_{r,+1}$; (**b**) comparison of transmission spectra showing a resonance wavelength shift due to change in RI of the target layer before/after bio-interaction. (dashed curve: before multivalent binding ($n_{h}$ = 1.34), solid curve: after multivalent binding ($n_{h}$ = 1.34, $n_{h,up}$ = 1.39)),$\;\Delta \lambda_{r}$: resonance wavelength shift, ΔT: transmission extinction.

Figure 3a shows the transmission spectrum of a grating waveguide having a target layer as shown in the inset of Figure 2 and the corresponding $\delta \lambda_{FWHM}$. The stopband is observed due to a strong feedback and the interference of the guided light in the grating waveguide core. Multiple resonance modes are created by the Fabry–Perot feedback. The +1 resonance mode ($\lambda_{r,+1}$), which is located on the longer wavelength side of the stopband, is used as the detecting mode because FWHM reaches its minimum at the mode when compared with other modes. Although the resonance mode located on the shorter wavelength side of the stopband, $\lambda_{r,-1}$, also exhibits a narrow FWHM similar to that at $\lambda_{r,+1}$, we utilized the $\lambda_{r,+1}$ mode as the detecting mode. It is because the resonance wavelength typically shifts toward the direction of the longer wavelength when the RI of the target material such as an aqueous solution or a functional hydrogel layer increases because of some biochemical phenomenon. If the $\lambda_{r,-1}$ mode is used as a detecting wavelength, the transmission profile of the next resonance mode $\lambda_{r,-2}$, which is located next to the $\lambda_{r,-1}$ mode, may possibly overlap the original target wavelength $\lambda_{r,-1}$. The overlap of these two resonance profiles before and after the sensing process may degrade the overall sensor performance. Figure 3b shows the transmission spectrum and its shift due to the change in RI of the functional hydrogel layer after multivalent binding of proteins. The dashed curve exhibits the transmission spectrum when the RI of a functional hydrogel layer is 1.34 before multivalent binding ($n_{h}$ = 1.34). The solid curve exhibits the transmission spectrum when the RIs of the upper and lower hydrogel layer become 1.39 and 1.34, respectively ($n_{h,up}=1.39,\;n_{h}$ = 1.34). It should be noted that the amount of change in RI is relatively large because the RI of the functional hydrogel layer can be made to change significantly to sense the multivalent binding effectively [26]. The resonance wavelength shift $\Delta \lambda_{r}$ is calculated as 0.62 nm and the corresponding transmission extinction ΔT as 22.82 dB.

## 3. Simulation of Dependence of Performance Evaluation on Grating Parameters

### 3.1. Duty Ratio Dependence

**Figure 4:**
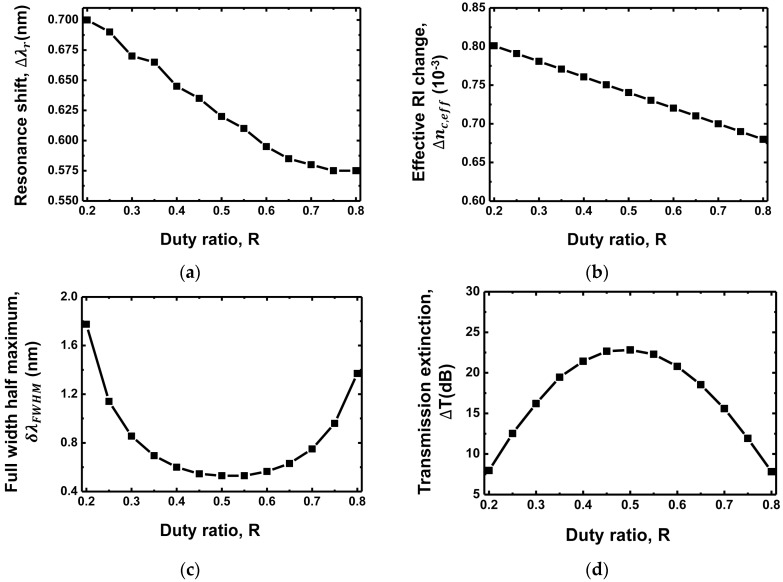
(**a**) Resonance shift ($\Delta \lambda_{r}$); (**b**) Effective change in RI ($\Delta n_{eff}$); (**c**) FWHM ($\delta \lambda_{FWHM}$); (**d**) Transmission extinction (ΔT) as functions of the duty ratio of the grating waveguide.

Figure 4a,b shows the resonance shift ($\Delta \lambda_{r}$) and the effective RI change ($\Delta n_{eff}$) as functions of duty ratio (R = $\frac{W_{t}}{\Lambda }$) of the grating structure, respectively. The duty ratio varies from 0.2 to 0.8. It is noted that the RI of the functional hydrogel layer changes in accordance with the double layer model as shown in Figure 2, assuming multivalent binding detection [19]. The resonance shift $\Delta \lambda_{r}$ becomes smaller when the duty ratio increases. It is 0.7 nm for the duty ratio of 0.2 and 0.575 nm for the duty ratio of 0.8. This is because the evanescent optical field in the target hydrogel layer decreases with the decrease in the grating groove width $W_{g}\;\left(=\Lambda -W_{t}\right)$. Because the amount of evanescent field decreases with the duty ratio, the RI of the hydrogel layer and the effective change in RI of the waveguide core $\Delta \;n_{c,eff}$ decrease correspondingly, as shown in Figure 4b. This result corresponds to [16]

(2) $$\Delta \;n_{c,eff}=\left(1-R\right)\Delta n_{g,eff}+\;R\Delta n_{t,eff},$$

where $\Delta n_{g,eff}$ is the effective change in RI of the grating groove width and $\Delta n_{t,eff}$ is the effective change in RI of the grating tooth width. Figure 4c shows the FWHM at $\lambda_{r,+1}$. The FWHM becomes small as the duty ratio approaches 0.5. This is because the narrowest FWHM can be achieved when the phase difference (=path difference) between the feedback light at the tooth-groove and groove-tooth interfaces is minimized. The FWHM is 0.53 nm at the duty ratio of 0.5. Based on the resonance shift and FWHM results, we can evaluate the transmission extinction performance ΔT as shown in Figure 4d. The transmission extinction, which reflects both the effect of the resonance shift and the FWHM, exhibits an exactly reverse trend to the result of FWHM in Figure 4c. The FWHM performance affects the transmission extinction more dominantly than the resonance shift performance in this case. Therefore, the determination of the duty ratio to achieve a narrower FWHM than to achieve a larger resonance shift is preferable for the design of a high-performance grating waveguide sensor. 

### 3.2. Grating Period Dependence

**Figure 5:**
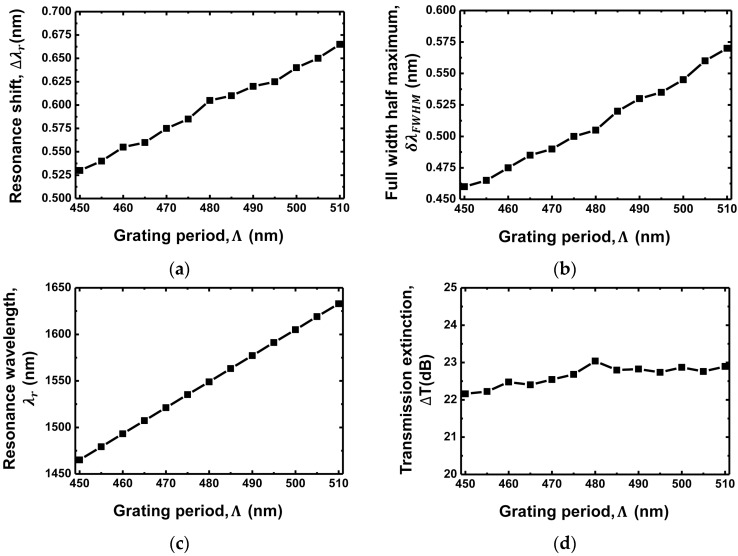
(**a**) Resonance wavelength shift ($\Delta \lambda_{r}$; (**b**) FWHM ($\delta \lambda_{FWHM}$); (**c**) Resonance wavelength ($\lambda_{r}$); (**d**) Transmission extinction (ΔT) as functions of the grating period of the grating waveguide.

Figure 5a shows the resonance shift $\Delta \lambda_{r}$ as a function of the grating period Λ. The resonance shift is 0.53 nm for Λ = 450 nm and increases to 0.665 nm for Λ = 510 nm. A longer grating period exhibits a larger resonance shift. It can be explained as follows: the center of the stopband, Bragg wavelength ($\lambda_{B}$), satisfies the following relationship with the grating period Λ and effective RI of the waveguide core $n_{c,eff}$ [29].

(3) $$\Delta \lambda_{B}=2\Lambda \Delta n_{c,eff}$$

Because the stopband width is maintained constant when an etching depth is fixed, the resonance wavelength shift becomes larger with the increase in the grating period [29]. Figure 5b,c shows the FWHM and the resonance wavelength $\lambda_{r}$ as functions of the grating period Λ, respectively. The FWHM becomes larger as the grating period increases. It is 0.46 nm for Λ = 450 nm and increases to 0.57 nm for Λ = 510 nm. This is because the resonance wavelength increases with the grating period, as shown in Figure 5c. The shorter resonance wavelength provides a stronger feedback in the waveguide, which results in the narrower FWHM. The resonance wavelength is 1465.11 nm for the grating period of 450 nm, whereas it increases to 1632.9 nm for the grating period of 510 nm. It can also be confirmed by (1). Figure 5d shows the transmission extinction as a function of the grating period. Its variation is maintained within the 1-dB range, exhibiting no significant performance variation with the change in the grating period because of the trade-off between the resonance shift and the FWHM performance. 

### 3.3. Etching Depth Dependence

**Figure 6:**
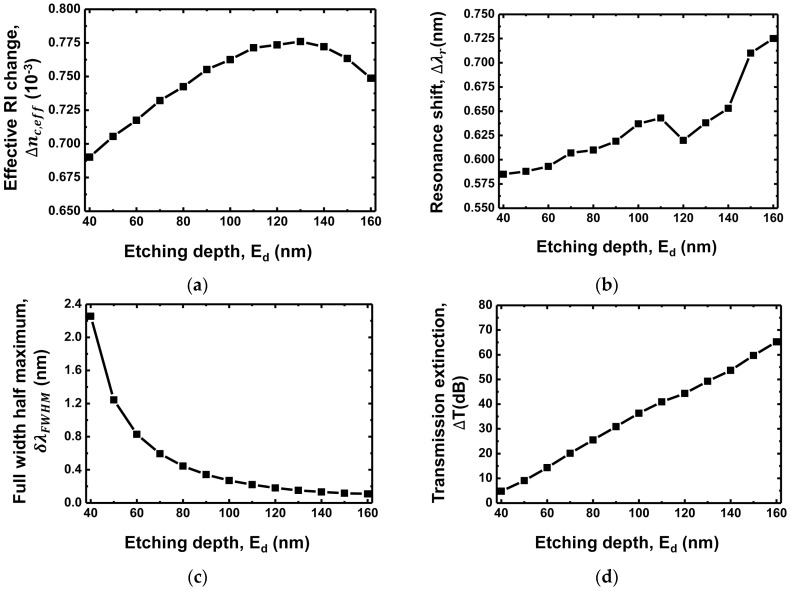
(**a**) Effective RI change ($\Delta n_{eff}$); (**b**) Resonance shift ($\Delta \lambda_{r}$); (**c**) FWHM ($\delta \lambda_{FWHM}$); (**d**) Transmission extinction (ΔT) as functions of the etching depth of the grating waveguide.

Figure 6a shows the effective change in RI $\Delta n_{c,eff}$ as a function of the etching depth $E_{d}$. It increases with the increase in the etching depth up to approximately 130 nm, whereas it decreases as the etching depth increases above 130 nm. Figure 6b shows the resonance shift $\Delta \lambda_{r}$ as a function of the etching depth $E_{d}$. The resonance shift is 0.585 nm for $E_{d}$ = 40 nm and increases to 0.725 nm for $E_{d}$ = 160 nm. The thickness of the waveguide core $t_{c}$ is set at 275 nm. The evanescent optical field in the target layer becomes stronger as the etching depth increases, resulting in the increase in the resonance frequency shift. Typically, the resonance shift $\Delta \lambda_{r}$ and the effective RI change $\Delta n_{c,eff}$ exhibit similar trend with the variation in the grating period, as shown in Figure 4a,b, because both are mainly affected by the amount of the evanescent optical field. On the other hand, the resonance shift $\Delta \lambda_{r}$ and the effective RI change $\Delta n_{c,eff}$ exhibit a slightly different trend with the variation in the etching depth, as shown in Figure 6a,b. This is because the etching depth also affects the stopband width, which changes the resonance shift, as shown in Figure 5b. Figure 6c shows the FWHM as a function of the etching depth $E_{d}$. The FWHM is 2.281 nm for $E_{d}$ = 40 nm, and it significantly reduces to 0.125 nm for $E_{d}$ = 160 nm. The deep etching causes strong feedback of the guided light in the grating structure, resulting in the narrow FWHM. As a result, we obtain the increase in the transmission extinction of 65.2 dB with the deep etching of 160 nm.

**Figure 7:**
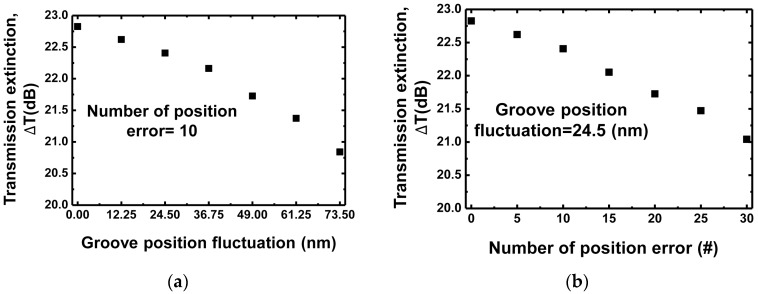
(**a**) Effect of groove position fluctuation on transmission extinction ∆T; (**b**) Effect of the number of error positions on ∆T. The grating period is 490 nm, the etching depth is 75 nm, and the duty ratio is 0.5.

Any fluctuations in the grating structures—such as groove size and position—after device fabrication, may affect the performance of the sensor designed using the optimized parameters. Figure 7a shows the effect of groove position fluctuation on the sensor performance, transmission extinction ΔT. The grating period is 490 nm, the etching depth is 75 nm, and the duty ratio is 0.5. The number of the error positions of the groove are 10 (=5% of the total 200 gratings). These parameters have been chosen without careful parameter optimization, which is yet to be discussed in Section 4. The transmission extinction ΔT decreases with an increase in position error, exhibiting 2.0 dB degradation for a position fluctuation of 73.5 nm. Figure 7b shows the effect of the number of error positions on ΔT. The error from the original error-free position is 24.5 nm (=5% of a grating period 490 nm). The transmission extinction ΔT decreases with an increase in the amount of the position error, exhibiting 1.7 dB degradation when using 30 position errors. It will be an interesting future study to evaluate the sensor performances and optimization procedure dependencies on the possible fluctuations of sensor structures and target biological environment.

## 4. Discussions

We calculated and analyzed the resonance shift, FWHM, and the resultant transmission extinction, as functions of the three major design parameters of the grating waveguide, which are duty ratio, grating period, and etching depth. We found that among the three parameters, the etching depth has the greatest effect on the transmission extinction performance of the grating waveguide sensor. The results and analysis are summarized in Table 1 and described as follows. 

The maximum transmission extinction is achieved at the duty ratio of approximately 0.5. The transmission extinction performance does not change significantly with the variation in the grating period. Although the grating period does not affect the transmission extinction performance, its control can be utilized to set the resonance wavelength of the grating waveguide. The increase in the etching depth enhances the resonance shift as well decreases the FWHM, resulting in significant improvement in the transmission extinction performance. However, the increase in the etching depth might be limited by the fabrication-related waveguide scattering loss. The determination procedure of the grating structure design for obtaining the maximum transmission extinction can be summarized as follows. Set the duty ratio as 0.5; Set the acceptable etching depth $E_{d}$ as deep as possible considering fabrication issues and waveguide loss; and Match the exact target wavelength of the specific sensor application with the sensor resonance wavelength by proper grating period control. 

**Figure 8:**
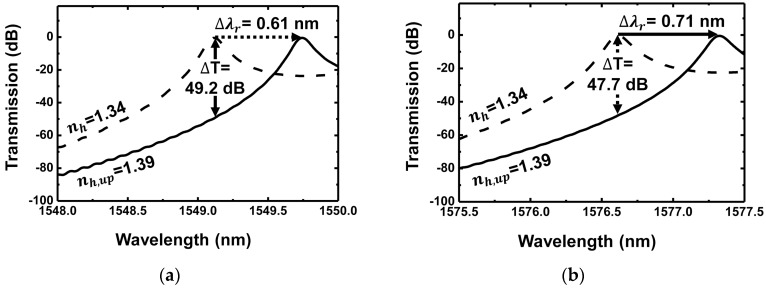
Transmission spectrum by applying the optimized grating waveguide parameters to achieve maximum (**a**) transmission extinction; (**b**) resonance shift before and after bio-interaction detection in the target layer. (Dashed curve: before multivalent binding ($n_{h}$ = 1.34), solid curve: after multivalent binding ($n_{h,up}$ = 1.39).

Figure 8a shows the transmission spectrum before and after target detection using the design rules and parameters discussed when the same target layer as in Figure 2 is used. The grating parameters are determined to achieve maximum transmission extinction. Figure 8a shows the resonance shift of 0.61 nm, FWHM of 0.185 nm, and resultant transmission extinction of 49.2 dB. It exhibits improvement in the transmission extinction of more than 26 dB compared with the result in Figure 8b, where the careful selection of the grating parameters is not considered. Figure 8b shows the transmission spectrum to achieve the maximum resonance shift. The measurement of the resonance shift sometimes requires lower-cost equipment and simple configuration than the measurement of the transmission extinction at a specific wavelength [18]. Our design rule and procedure also provide a large resonance shift of 0.71 nm when compared with the result in Figure 3b. The sensitivity presenting a resonance shift performance is 165.0 nm/RIU for the sensor with the design parameters as shown in Figure 8b, where RIU stands for an RI unit. This is a 17.8% improvement compared with the experimental results in [14]. 

## 5. Conclusions

We performed a theoretical performance evaluation of the silicon-based grating waveguide, which is an effective method to achieve high performance of biochemical sensors. We considered both the resonance wavelength shift and FWHM of the transmission spectrum and set the transmission extinction as the major FOM to characterize the RI sensor performance. The transmission extinction performance was evaluated by considering the three major parameters comprising the grating structure, duty ratio, grating period, and etching depth. We found that the etching depth plays the most significant role in achieving the maximum transmission extinction. We summarized the relationships of the three parameters with the resonance wavelength shift and FWHM as well as the transmission extinction. By means of our analysis, we successfully achieved the optimization of the grating structure parameters for the silicon-based grating waveguide sensor. The analysis and the design guideline would enable the widespread use of the grating waveguide sensor for high-performance and low-cost biochemical sensors. 

## Figures and Tables

**Table 1:** Relationship between grating waveguide parameters and transmission extinction performance.

Grating Waveguide Parameters	Resonance Shift ($\Delta \lambda_{r}$)	FWHM ($\delta \lambda_{FWHM}$)	Resonance Wavelength ($\lambda_{r}$)	Transmission Extinction (ΔT)
Duty ratio (R)	Proportional decrease	Quadratic (min. at 0.5)	Proportional increase	Quadratic (max. at 0.5)
Grating period (Λ)	Proportional increase	Proportional increase	Proportional increase	Almost constant
Etching depth ($E_{d}$)	Proportional increase	Exponential decrease	Proportional decrease	Proportional increase
